# Down-regulation of kallikrein-related peptidase 5 (*KLK5*) expression in breast cancer patients: a biomarker for the differential diagnosis of breast lesions

**DOI:** 10.1186/1559-0275-8-5

**Published:** 2011-05-31

**Authors:** Margaritis Avgeris, Georgia Papachristopoulou, Athanasios Polychronis, Andreas Scorilas

**Affiliations:** 1 Department of Biochemistry and Molecular Biology, University of Athens, Panepistimiopolis, 15701 Athens, Greece; 2 Oncological Hospital "G.Gennimatas" IKA-ETAM of Athens, Asopiou 4, 11473 Athens, Greece

**Keywords:** KLK5, Breast Cancer, Cancer Biomarkers, Tumor Markers, KLKs, Kallikreins, Serine Proteases, Proteolysis

## Abstract

**Background:**

Kallikrein-related peptidase 5 (KLK5) is a secreted trypsin-like protease of the KLK family, encoded by the *KLK5 *gene. KLK5 has been found to cleave various extracellular matrix components, as well as to activate several other KLK proteases, triggering the stimulation of tissue microenvironment proteolytic cascades.

**Material and Methods:**

*KLK5 *expression levels were quantified in 102 cancerous and benign breast tissue specimens, obtained by randomly chosen patients, using RT-qPCR assay. Subsequently, advanced biostatistics were applied in order to analyze the *KLK5 *expression profile in the two patients' cohorts and also to evaluate its clinical significance for the discrimination of breast tumors.

**Results:**

A statistically significant (p < 0.001) down-regulation of the *KLK5 *expression levels were observed in the malignant specimens compared to the benign ones. Logistic regression and ROC curve analysis revealed the significant (p < 0.001) and the independent (p < 0.001) value of the *KLK5 *expression quantification, for the discrimination of the malignant from the benign mammary gland biopsies. Moreover, *KLK5 *expression levels correlate with the pre-menopausal status (p < 0.005) as well as the ER-negative staining (p = 0.028) of women with breast cancer.

**Conclusions:**

The quantification of *KLK5 *expression in breast tissue biopsies may be considered as a novel and independent biomarker for the differential diagnosis between malignant and benign tumors of the mammary gland.

## Introduction

The central role of proteases is widely proven in the complex and multiparametric scene of cancer development and progression. Their abnormal expression and function leads mainly to a deregulated degradation of extracellular matrix (ECM) components, as well as to the activation of several signaling molecules and biochemical pathways. Hence, proteases contribute at first to the creation of a malignant phenotype as well as an impending invasiveness facilitating the spread of the tumor cells. Among the proteases of the human degradome, tissue kallikrein 1 (KLK1) and the kallikrein-related peptidases (KLK2-KLK15) compose a group of 15 conserved secreted serine proteases with trypsin- or chymotrypsin-like activities. They are encoded by 15 structurally homologous genes (*KLK1-KLK15*), which have been mapped on the 19q13.3-4 chromosomal region. KLKs comprise the largest family of proteases including all catalytic classes, as they are not interrupted by any non kallikrein-related protease gene [1].

Recent studies regarding the KLKs substrate specificity have revealed their significant association with the establishment and progression of the malignancy. These secreted proteases are implicated in proteolytic cascade pathways resulting to extended cleavage of ECM components. The ECM degradation and remodeling, mediated directly by KLKs or via the KLKs-induced activation of other extracellular proteases, interrupt ECM physical barriers and cells' interaction, facilitating angiogenesis and cancer cells' invasiveness and metastasis [2]. Moreover, during the early stages of the disease, KLKs influence the availability of growth factors and therefore regulate tumor cells proliferation. In particular, they were found to cleave insulin-like growth factor binding proteins (IGFBPs), thus releasing, in this way, the mitogenic role of insulin-like growth factors (IGFs) [3]. Furthermore, KLKs are able to stimulate protease-activated receptors (PARs) through the cleavage of the extracellular N-terminal segment. PARs activation results in the triggering of an intracellular biochemical cascade leading to mitogen-activated protein kinase (MAPK) activation and cell proliferation [3,4].

Deregulated expression and secretion of KLKs has been detected in numerous malignancies, especially in the endocrine-related ones. Beyond the crucial impact of KLKs function upon the origination and progression of cancer, a plethora of studies have already underlined their potential use as diagnostic and prognostic biomarkers, as well as their contribution to the cancer patients' monitoring [5,6]. This is clearly affirmed by the clinical use of prostate specific antigen (PSA)/KLK3, in male population screening, early diagnosis and monitoring of prostate cancer patients.

Nowadays, breast cancer represents the most frequently diagnosed carcinoma and the second leading cause of cancer-related deaths over female populations [7]. The early detection of breast neoplasia is beneficial for the successful treatment of the patients. However, breast tumors embrace a large number of benign lesions, requiring different clinical management in comparison to the malignant ones. Consequently, the screening among KLKs for new breast cancer-specific biomarkers could further add to the clinical diagnostic tools in the detection of mammary malignancies and the discrimination from non-cancerous lesions. Nevertheless, the accuracy of patients' prognosis will be improved and a novel panel of candidate markers will be created and evaluated.

Kallikrein-related peptidase 5 (KLK5) is a secreted trypsin-like serine protease, encoded by the *KLK5 *gene of the *KLKs *gene family, under the transcriptional control of estrogens and progestins [8,9]. The proteolytic activity of KLK5 has already been proven on a big number of ECM components, such as collagens, fibronectin and laminin [10]. KLKs are secreted as inactive zymogens (pro-KLKs) and their activation depends on the proteolytic cleavage of their N-terminus pro-peptide via autocatalysis or through other KLKs or non-KLK proteases [11]. The KLK5 autoactivation from the secreted pro-KLK5 represents an initial process of the KLKs proteolytic cascade, triggering the activation of several other KLKs (KLK2, -3, -6, -7, -11, -12 and -14) [12]. Therefore, KLK5 has been considered to be the regulator of KLKs extracellular proteolytic cascade, counterbalancing the molecular microenviroment between normal physiology and cancer. Therefore, *KLK5 *expression quantification is critical for both the discovery of the early cellular and molecular alterations in breast cancer cells, as well as for the identification of novel diagnostic and prognostic biomarkers.

Apart from breast cancer, the prognostic value of *KLK5 *expression has already been demonstrated for ovarian, bladder, prostate, colorectal and testicular cancer. In ovarian cancer, overexpression of KLK5 protease in tumor tissues is associated with more advanced stages and grade of the disease, as well as shorter DFS and OS of the patients [13]. In addition, the KLK5 concentration in serum was reported to correlate with ovarian cancer patients' poor response to chemotherapy [14], and poor outcome [15]. In agreement with the aforementioned significance of KLK5 for the ovarian cancer patients' prognosis, higher *KLK5 *gene transcriptional levels were detected in patients with aggressive forms of epithelial ovarian tumors [16], while the differential profile of the expressed *KLK5 *mRNA transcript variants between normal and malignant ovarian cancer cells was uncovered [17]. In bladder cancer, upregulated *KLK5 *expression was recently found to correlate with the invasive phenotype of the disease [18]. On the other hand, higher levels of *KLK5 *expression were detected more frequently in less aggressive forms of prostate tumors [19,20], while recent findings have demonstrated the clinical use of *KLK5 *expression as a biomarker for the prediction of prostate patients' response to chemotherapy [21,22]. In addition, an unfavorable patients' outcome has been associated with the KLK5 protease accumulation in colorectal cancer tissues [23]. Finally, in testicular cancer, the down-regulation of *KLK5 *gene expression in tumor tissues is associated with later stage and more invasive tumors [24].

Considering this significant and central role of KLK5, we used a quantitative real-time RT-PCR (RT-qPCR) assay for the *KLK5 *expression quantification in 102 malignant and benign tumor breast tissues. Extensive statistics were applied in order to investigate the *KLK5 *expression association with the histological features of breast cancer patients, aiming to examine its clinical value for the discrimination of the cancerous from non-cancerous breast lesions.

## Materials and methods

### Breast tissue specimens and study population

A total of 69 malignant and 33 benign tumor breast tissue specimens were collected in order to analyze the *KLK5 *expression profile in breast tumors. The patients, having undergone surgical tumor dissection between October 2005 and May 2009, had not received any hormonal therapy or chemotherapy treatment prior to the surgery. Histological analysis was performed, by the same pathologist, for the discrimination of the malignant from the benign tumor specimens, as well as for the evaluation of breast cancer patients' TNM stage, grade, estrogen receptor (ER) and progesterone receptor (PR) staining, axillary lymph node status and tumor size. The 69 breast cancer patients were diagnosed with primary ductal adenocarcinoma, while among the 33 patients carrying benign lesions, 22 (69.7%) were suffering from fibroadenoma, 5 (15.2%) from fibrocyst, 4 (12.1%) from fibroepitheliosis and 1 (3%) from tubular adenoma.

Our study was performed with respect to the ethical standards of the 1975 Declaration of Helsinki, as revised in Tokyo 2004. Institutional approval for the use of the breast samples was obtained from the ethical committee of the Oncological Hospital "G.Gennimatas" IKA-ETAM of Athens.

### Total RNA extraction and cDNA synthesis

Total RNA was isolated from 50-100 mg breast tissue samples using TRI-reagent (Ambion Inc., Austin, TX, USA) following the manufacturer's instructions. Total RNA concentration and quality were determined spectrophotometrically at 260 and 280 nm, while RNA integrity was evaluated using agarose gel electrophoresis. Reverse transcription of the mRNA molecules into first-strand cDNA, was carried out using 1 μg of total RNA from each tissue specimen, M-MuLV Reverse Transcriptase RNase H^- ^(Finnzymes Oy, Espoo, Finland) and an Oligo(dT) oligonoucleotide as reverse transcription primer.

### Quantitative Real-Time PCR

A SYBR-Green fluorescence-based quantitative Real-Time PCR assay (qPCR) was used for the determination of *KLK5 *expression levels in the breast specimens on an ABI Prism 7500 Thermal Cycler (Applied Biosystems, Foster City, CA, USA). Based on the mRNA sequences from the NCBI Sequence database, gene specific primers were designed and synthesized for *KLK5 *target gene (NCBI Reference Sequence: NM_012427.4) and *HPRT1 *(hypoxanthine phosphoribosyltransferase-1) endogenous reference gene (NCBI Reference Sequence: NM_000194.2) using the Primer Express software (Applied Biosystems). A 151bp fragment was amplified using the *KLK5 *forward 5'-CCGGTGACAAAGCAGGTAGAG-3' and reverse 5'-GTGAACTTGCAGAGGTTCGTGTA-3' pair of primers, whereas the use of the *HPRT1 *forward 5'-TGGAAAGGGTGTTTATTCCTCAT-3' and reverse 5'-ATGTAATCCAGCAGGTCAGCAA-3' primers led to the accumulation of a specific 151bp product.

The final 10 μL reaction volume includes 5 μL of 2 × Power SYBR^® ^Green PCR Master Mix (Applied Biosystems), 50 nM of each primer and 0.2 μL cDNA. The thermal protocol consists of a 10 min polymerase activation step at 95°C, followed by 40 cycles of denaturation at 95°C for 15 sec and primer annealing and extension at 60°C for 1 min.

Both the *KLK5 *target gene and the *HPRT1 *reference gene sequences were amplified in separate duplicate reactions for each sample and the average C_T _value was calculated. Relative quantification analysis, using the comparative C_T _(2^-ΔΔCT ^) method [25], was completed for the determination of the *KLK5 *mRNA expression, where the *HPRT1 *expression was used as the endogenous reference gene for the normalization of the *KLK5 *expression and the BT20 breast cancer cell line as a calibrator, as previously described^26^.

### Statistical Analysis

Due to the fact that the distributions of *KLK5 *expression levels in breast tumors were not Gaussian, the analysis of the differences in the two groups of patients was performed with the non-parametric Mann-Whitney U test. The ability of the variables to predict the presence of breast cancer was studied using univariate and multivariate unconditional logistic regression analyses. Associations between *KLK5 *expression status and other clinicopathological variables of the breast cancer patients were analyzed using the Chi-Square (χ2) and Fisher's exact tests, where appropriate.

Receiver operating characteristic (ROC) curve was constructed for *KLK5 *expression levels, by plotting sensitivity versus (1-specificity). The areas under the ROC curves (AUC) were analyzed by the Hanley and McNeil method.	A p-value of 0.05 (or less) was considered as statistically significant.

## Results

### Determination of the RT-qPCR amplification efficiencies for *KLK5 *and *HPRT1*

**Figure 1:**
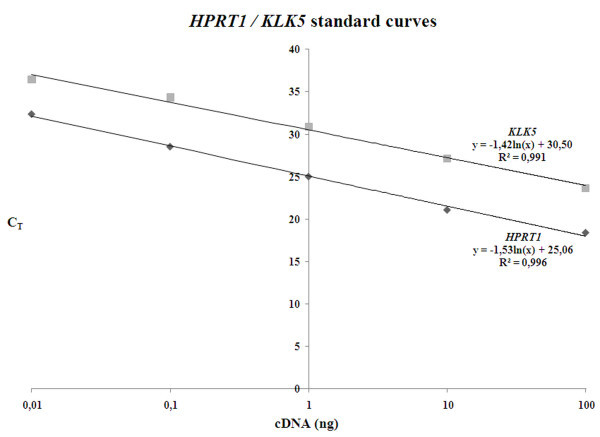
**Relative quantification of *KLK5 *expression in breast specimens by SYBR Green fluorescence-based Real-Time PCR**. Separate calibration curves for *KLK5 *and *HPRT1 *expression were constructed from serial dilutions of BT20 breast cancer cells' total cDNA.

The relative quantification of the *KLK5 *mRNA expression was performed with the use of the comparative C_T _(2^-ΔΔCT^) method, whereas the *HPRT1 *was employed as endogenous reference gene and the BT20 breast cancer cell line as the calibrator of the assay. However, the use of 2^-ΔΔC^_T _method requires the approximately equal amplification efficiencies of the target and the reference genes. Therefore, prior to the assessment of the *KLK5 *expression levels, we designed a validation experiment using as template serial dilutions of the BT20 cDNA covering five orders of magnitude (0.01, 0.10, 1.00, 10.0 and 100 ng) in a single run. Plotting the C_T _versus the log of the cDNA mass of the input amount we have assessed the C_T _values' linear increase of the *HPRT1 *amplification (y = -1,53ln(x) + 25,06) and the *KLK5 *amplification (y = -1,42ln(x) + 30,50) (Figure 1), and determined the *HPRT1 *and *KLK5 *amplification efficiencies, which was 91.6% and 102.0%, respectively. The approximately equal amplification efficiencies of the *KLK5 *and *HPRT1 *genes allow the quantification of the *KLK5 *expression levels of the breast tissues with the 2^-ΔΔCT ^relative quantification method.

### *KLK5 *expression was found to be downregulated in the malignant breast tissues

**Figure 2:**
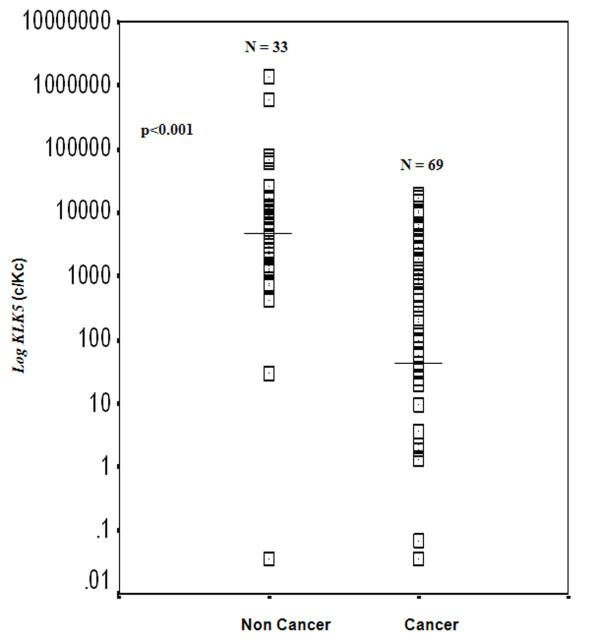
**Distribution of *KLK5 *mRNA expression levels of the cancerous and the non-cancerous breast tissue specimens**. Horizontal lines indicate the median (50^th ^percentile) *KLK5 *expression. The p value was calculated using the Mann-Whitney U test.

The *KLK5 *expression analysis in the collected breast tissues revealed a statistically significant (p < 0.001) down-regulation of the gene transcription levels in the malignant tumor specimens compared to the benign ones (Figure 2). The *KLK5 *expression of the benign tissue specimens ranged between 0.035-1380.4×10^3 ^*KLK5 *mRNA copies/10^3 ^*HPRT1 *mRNA copies (c/Kc), while the expression in the tissue specimens obtained by the breast cancer patients varied between 0.035-19.6x10^3 ^c/Kc. The median (50^th ^percentile) value of the *KLK5 *expression (41.5 c/Kc) in the cancer patients' specimens was found to be approximately 105-fold decreased, compared to that found in the non-cancer patients' breast tissues (median: 4374.1 c/Kc). The reduced *KLK5 *transcription in the malignant cells was also revealed by the breast cancer patients' *KLK5 *expression analysis in quartiles, compared to the same analysis of the control cohort of patients with benign breast modifications (Table 1).

**Table 1:** Descriptive statistics of *KLK5 *expression in breast tissues

			Quartiles				
Variables	Mean ± SE^a^	Range	10	25	50 (median)	75	90
Cancer (N = 69)*							
*KLK5 *(c/Kc)^b^	(1.67 ± 0.48) × 10^3^	0.035-19.6 × 10^3^	0.035	0.013	41.5	1129.3	5937.9
							
**Non Cancer (N = 33)**							
*KLK5 *(c/Kc)^b^	(71.1 ± 44.7) × 10^3^	0.035-1380.4 × 10^3^	180.6	1264.3	4374.1	12320.5	74702.3

* Ductal histotype

^a ^Standard error

^b ^*KLK5 *mRNA copies/10^3 ^*HPRT1 mRNA *copies normalized to the calibrator (BT20 breast cancer cell line)

### Estimation of the predictive value of *KLK5 *expression regarding the presence of breast cancer

**Figure 3:**
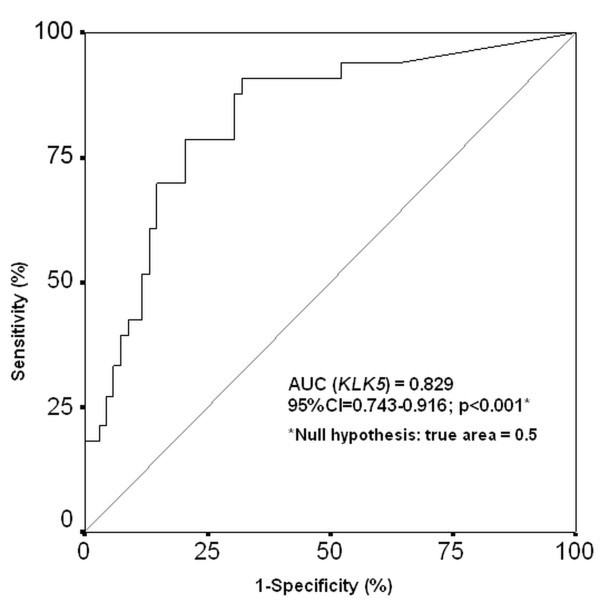
**ROC analysis of the *KLK5 *expression quantification in breast cancer prediction**. The area under the ROC curves (AUC) was analyzed by Hanley and McNeil method.

An extended statistical analysis, including logistic regression (Table 2) and ROC analysis (Figure 3), was carried out in order to evaluate the clinical value of the *KLK5 *expression down-regulation for the prediction of breast cancer and its discrimination from non-cancer breast lesions in tissue biopsies. The univariate logistic regression analysis clearly shows the statistically significant (p < 0.001) negative correlation between the *KLK5 *expression levels and the risk of a patient to suffer for breast cancer. In particular, patients with low *KLK5 *expression levels are at a higher risk for breast malignancy as compared to those with high *KLK5 *expression profiles (Log_10_*KLK5 *HR: 0.47, 95% CI: 0.32 - 0.68). The differential diagnostic impact of the *KLK5 *expression levels between the malignant and the benign breast tumors as well as the use of the *KLK5 *expression analysis for the prediction of breast cancer was also assessed by ROC analysis. A statistically significant (p < 0.001) ability of the *KLK5 *expression analysis to distinguish breast cancer patients from those bearing non-cancerous breast alterations, was illustrated by the increased AUC (AUC = 0.829; 95%CI = 0.743-0.916).

**Table 2:** Logistic Regression Analysis for Predicting the Presence of Breast Cancer

	*Univariate Analysis*			*Multivariate Analysis*		
**Covariant**	**Crude Odds Ratio**	**95% CI**	p-value*	**Crude Odds Ratio**	**95% CI**	p-value*

*Log KLK5*	0.47	0.32 - 0.68	< 0.001	0.42	0.26 - 0.67	< 0.001
Size	1.12	0.80 - 1.57	0.52	1.13	0.44 - 3.42	0.69
Age	1.10	1.06 - 1.15	0.001	1.12	1.06 - 1.18	0.001

CI: Confidence Intervals

*Test for trend

A multivariate logistic regression analysis was adjusted to Log_10_*KLK5 *expression levels, the tumor size and the age of the patients (Table 2) with the intention to examine the independence of the differential diagnosis potential of the *KLK5 *mRNA expression between the malignant and benign breast specimens. The statistically significant (p < 0.001) negative correlation of the *KLK5 *mRNA levels and the risk for breast cancer (crude odds ratio: 0.42, 95% CI: 0.26 - 0.67) in relation to the patients' age and tumor size demonstrates the independent ability of *KLK5 *expression quantification to discriminate the malignant breast tumor cases from those with benign lesions of the mammary gland.

### Association of the *KLK5 *expression with the clinicopathological features of the breast cancer patients

Following the estimation of the *KLK5 *expression analysis clinical value for the differential diagnosis of breast malignancies from non-cancerous lesions in breast tissue biopsies, we further examined the association of the *KLK5 *expression with the clinicopathological variables of the breast cancer patients (Table 3). An optimal cut-off value equal to the 70^th ^percentile of the *KLK5 *expression levels was adopted in order to separate the patients in *KLK5-*negative and *KLK5-*positive cohorts. Statistically strong associations were observed between the *KLK5 *expression and the menopausal status (p = 0.005), as well as the estrogen receptor (ER) staining (p = 0.028) of the breast cancer patients.

**Table 3:** Associations between *KLK5 *status and the clinicopathological variables of the breast cancer patients

		No. of patients (%)		
Variable	Total	*KLK5*- negative *KLK5*- positive		p value
Menopausal status*				
Pre/peri	54	22 (40.7)	32 (59.3)	0.005^b^
Post	47	33 (70.2)	14 (29.8)	
				
Grade^c^				
I	3	1 (33.3)	2 (66.7)	
II	30	21 (70.0)	9 (30.0)	0.34^d^
III	34	25 (73.5)	9 (26.5)	
				
TNM Tumor size				
<2 cm	26	17 (65.4)	9 (34.6)	
2-5 cm	37	27 (73.0)	10 (27.0)	0.79^d^
> 5 cm	4	3 (75.0)	1 (25.0)	
				
ER status				
Negative	16	7 (43.8)	9 (56.3)	0.028^b^
Positive	51	39 (76.5)	12 (23.5)	
				
PR status				
Negative	24	15 (62.5)	9 (37.5)	0.42^b^
Positive	43	31 (72.1)	12 (27.9)	

^a ^Cutoff point: 820 c/Kc equal to 70^th ^percentile

^b ^Fisher's Exact Test

^c ^Bloom-Scarff-Richardson grading system

^d ^χ^2 ^test

^c^TNM system

* In the total studied population

In more details, post-menopausal breast cancer women displayed a significant downregulated *KLK5 *expression profile, with 70.2% of them to be classified as *KLK5-*negative, compared to the pre-/peri-menopausal ones, of whom only the 40.2% of them were categorized as *KLK5-*negative. The study of the association between the *KLK5 *expression and the ER status revealed a strong negative correlation in the breast cancer patients' cohort. The 76.5% of the ER-positive patients were classified as *KLK5*-negative, while the percentage of ER-negative patients being also *KLK5-*negative was only 43.8%. The associations between *KLK5 *expression and the breast cancer patients' histological grade, tumor size and PR staining were not proven to be statistically significant.

## Discussion

Deregulated expression of several KLKs has been detected in a large number of human malignancies, during the establishment and progression of the disease. These alterations are considered to constitute a fundamental feature of carcinogenesis, facilitating tumor cell growth and spread [2,3]. In addition, numerous data have prompted the expression analysis of KLKs as novel tumor biomarkers for the disease detection and the monitoring of cancer patients [5,6]. Focusing on breast neoplasia, the early differential diagnosis of the malignant from the non-cancerous cases is essential for the detection of breast cancer at an initial stage, as well as the decision of their appropriate treatment. The potential use of KLKs for this clinical need has already been proven for the *KLK4 *and *KLK14 *[26,27]. In the present study, we have analyzed the *KLK5 *expression levels in malignant and benign breast tissues, with the intension to assess its clinical value for the discrimination of the cancerous from the non-cancerous breast tumors and its association with the prevalence of the disease.

The *KLK5 *expression analysis highlights the gene's high transcriptional levels in mammary gland; where both the cancerous and the benign breast tissue specimens showed detectable *KLK5 *expression. In addition, the analysis revealed a statistically significant (p < 0.001) down-regulation of the gene's expression in the breast cancer patients' tissues, in relation to those obtained from patients with benign lessions. The gene transcription reduction is strongly depicted by the approximately 105-fold drop of the median *KLK5 *expression levels in cancerous specimens compared to non-cancerous ones. This significant down-regulation of *KLK5 *expression in malignant breast tissues has also been reported previously [28]. Moreover, the down-regulation of the *KLK5 *expression was also confirmed in breast cancer metastases compared to primary cancer cores, thus highlighting the downregulation of the *KLK5 *expression throughout breast cancer progression.

The clinical ability of the *KLK5 *expression quantification to discriminate the malignant from the benign lesions of the mammary gland was evaluated by both logistic regression and ROC curve analyses. The univariate logistic regression model disclosed a statistically significant (p < 0.001) elevated risk of the patients with reduced *KLK5 *expression to suffer from breast cancer. In addition, the ROC analysis illustrated the statistically significant (p < 0.001) value of the *KLK5 *expression quantification for the discrimination of the malignant from the benign specimens. Moreover, the multivariate logistic regression models supported the notion that *KLK5 *expression levels can independently predict the presence of breast cancinoma. Indeed, the fact that *KLK5 *expression was shown to constitute a tumor size-independent biomarker for breast cancer prediction highlights its significance for the discrimination of malignant lesions at an early stage of the disease.

The down-regulation of the *KLK5 *expression in breast neoplasia does not confirm previous studies, which showed elevated concentration levels of the KLK5 protein in the serum of breast cancer patients, compared to those bearing benign tumors [29]. However, the disruption of the breast tissue microenvironment architecture occurring during cancer establishment and progression leads to an extended leak of secreted extracellular molecules into blood circulation, causing their higher serum concentrations. Taking into consideration that KLK5 proteolytic function is responsible for the catalytic cleavage and activation of several others KLKs, the down-regulation of the *KLK5 *expression in breast cancer tissues possibly signifies the result of a negative regulatory mechanism. With this mechanism, the cells strive maybe to counterbalance the abnormal activation of the KLK proteolytic cascade contributing to their homeostasis.

Apart from the evaluation of *KLK5 *expression for the differential diagnosis of breast cancer, we further examined the *KLK5 *expression status association with breast cancer patients' clinocopathological features, in order to assess its prognostic significance for the patients. Statistically significant association of the *KLK5 *mRNA levels with the ER-negative staining (p = 0.028) and the pre-/peri-menopausal status (p = 0.005) was observed in breast cancer patients. The association between the *KLK5 *expression and the breast cancer patients' grade, tumor size and PR staining was not found to be statistically solid (p > 0.05). These results are in agreement with previous studies examining of the *KLK5 *expression prognostic value in breast cancer patients, whereas the *KLK5 *expression quantification proved to be an independent biomarker for the prediction of breast cancer patients' DFS and OS [30]. The association of the *KLK5 *expression levels with the pre-menopausal status of breast cancer patients was expected, due to the drop of circulating estrogens of the post-menopausal women. However, the correlation of the *KLK5 *expression with the ER-negative status of the cancer patients is in contrast with the up-regulation of *KLK5 *expression by estrogens. A possible explanation for this dissimilarity is the ER-independent regulated impact of estrogens on *KLK5 *transcription.

## Conclusion

The aim of the current study was the expression analysis of *KLK5 *in cancerous and benign breast lesions, targeting the evaluation of the clinical use of *KLK5 *expression for the discrimination of the malignant from the non-cancerous tumors of the mammary gland. Significantly decreased *KLK5 *transcription was detected in tissues of breast cancer patients compared to those bearing benign tumors. This down-regulation of *KLK5 *expression may be considered as an independent novel biomarker for the differential diagnosis between the malignant and benign breast tumors. A large scale *KLK5 *expression analysis in breast tissue specimens, as well the study of the *KLK5 *alternatively spliced variants must be carried out, in order to reinforce the significance of *KLK5 *expression analysis in breast cancer patients and to reveal the molecular alterations that occur during breast carcinogenesis.

## Competing interests

The authors declare that they have no competing interests.

## Authors' contributions

MA participated in the conception and design of the study, the acquisition, the analysis and the interpretation of the data, the statistical analysis and drafted the manuscript.

GP participated in the conception and design of the study and the acquisition, the analysis and the interpretation of the data.

AP participated in the conception and design of the study and the acquisition of the data.

AS participated in the conception and design of the study, the analysis and the interpretation of the data, the statistical analysis and supervised the study.

All authors read and approved the final manuscript.
